# Liquid biopsy techniques and lung cancer: diagnosis, monitoring and evaluation

**DOI:** 10.1186/s13046-024-03026-7

**Published:** 2024-04-01

**Authors:** Fei Ren, Qian Fei, Kun Qiu, Yuanjie Zhang, Heyang Zhang, Lei Sun

**Affiliations:** 1 Thoracic Surgery, The First Hospital of China Medical University, Shen Yang, 110000 China; 2 Department of Geriatrics, The First Hospital of China Medical University, Shen Yang, 110000 China; 3 Department of Oncology, Shengjing Hospital of China Medical University, Shen Yang, 110000 China; 4 Department of Hematology, The First Hospital of China Medical University, Shen Yang, 110000 China

**Keywords:** Lung cancer, Liquid biopsy, Circulating tumor cells, Circulating tumor DNA, Noncoding RNAs, Extracellular vesicles, Tumor metabolite, Tumor-educated platelet, Tumor-associated antigens

## Abstract

Lung cancer stands as the most prevalent form of cancer globally, posing a significant threat to human well-being. Due to the lack of effective and accurate early diagnostic methods, many patients are diagnosed with advanced lung cancer. Although surgical resection is still a potential means of eradicating lung cancer, patients with advanced lung cancer usually miss the best chance for surgical treatment, and even after surgical resection patients may still experience tumor recurrence. Additionally, chemotherapy, the mainstay of treatment for patients with advanced lung cancer, has the potential to be chemo-resistant, resulting in poor clinical outcomes. The emergence of liquid biopsies has garnered considerable attention owing to their noninvasive nature and the ability for continuous sampling. Technological advancements have propelled circulating tumor cells (CTCs), circulating tumor DNA (ctDNA), extracellular vesicles (EVs), tumor metabolites, tumor-educated platelets (TEPs), and tumor-associated antigens (TAA) to the forefront as key liquid biopsy biomarkers, demonstrating intriguing and encouraging results for early diagnosis and prognostic evaluation of lung cancer. This review provides an overview of molecular biomarkers and assays utilized in liquid biopsies for lung cancer, encompassing CTCs, ctDNA, non-coding RNA (ncRNA), EVs, tumor metabolites, TAAs and TEPs. Furthermore, we expound on the practical applications of liquid biopsies, including early diagnosis, treatment response monitoring, prognostic evaluation, and recurrence monitoring in the context of lung cancer.

## Introduction

As one of the deadliest and most common types of tumors in the world, lung cancer has become the leading cause of tumor-related deaths globally [1]. Pathologically, lung cancers are classified into small-cell lung cancers (SCLC) and non-small cell lung cancers (NSCLC). Of these, about 85% of lung cancers are NSCLCs, which include lung adenocarcinoma (LUAD) and lung squamous cell carcinoma (LUSC) [2]. Patients diagnosed with lung cancer typically exhibit advanced-stage disease, often accompanied by distant metastases at the onset of symptoms. This contributes significantly to the elevated mortality rates and unfavorable prognosis observed in patients with lung cancer [3]. Hence, identifying effective strategies for early screening and prompt tumor intervention represents a crucial approach to mitigating mortality in individuals diagnosed with primary lung cancer. Conventional diagnostic methods for lung cancer depend on techniques such as endoscopic ultrasonography-guided fine-needle aspiration (EUS-FNA), Magnetic Resonance Imaging (MRI), and low-dose spiral CT (LDCT), along with histopathological diagnostics [4, 5]. Nevertheless, each of these diagnostic methods comes with certain drawbacks, including invasiveness, elevated costs, radiation exposure to patients, a heightened risk of false positives, and potential surgical risks associated with obtaining tissue biopsies [6]. . Furthermore, despite ongoing exploration of the potential role of tumor biomarkers, there remains a dearth of dependable diagnostic biomarkers for lung cancer. Carcinoembryonic antigen (CEA) stands out as a widely employed clinical biomarker for lung cancer, holding significant diagnostic value in lung adenocarcinoma [7]. However, its lack of specificity poses a limitation, as it can indicate the presence of various tumors, including colorectal and breast cancers, as well as benign lung diseases [8, 9]. Consequently, relying solely on tumor biomarkers cannot furnish entirely accurate evidence for tumor diagnosis and necessitates evaluation in conjunction with diverse clinical symptoms, imaging tests, and other methodologies [10]. Indeed, it is highly conceivable that these modalities will have an adverse impact or burden on the patient.

Liquid biopsies have garnered widespread attention in recent years, primarily owing to their less invasive nature and the capability for frequent or continuous sampling, enabling the ongoing tracking of tumor progression and therapy response [11]. As tumor cells proliferate, liquid biopsies unveil additional insights into tumor characteristics by detecting tumor cells or genetic material released into bodily fluids [12]. This highly sensitive technique not only diagnoses tumors but also provides crucial details about the tumor, such as pathological typing, the stage of tumor progression, and mutations in tumor-associated genes [13]. While blood remains the most commonly utilized liquid biopsy in clinical practice, other clinical samples like cerebrospinal fluid, saliva, ascites, pleural fluid, and urine are also employed. Diverse sample sources contribute to revealing characteristics specific to different tumor types [14]. Presently, circulating tumor cells (CTCs), circulating tumor DNA (ctDNA), non-coding RNAs (ncRNA), microRNAs (miRNAs), tumor-associated antigens (TAA) and extracellular vesicles (EVs) stand as potential targets for liquid biopsy due to their ability to provide information about tumor genomics, transcriptomics, and proteomics [11, 15]. Liquid biopsy has proven highly beneficial in managing lung cancer, encompassing applications in early diagnosis, treatment strategies, therapy response monitoring, recurrence monitoring, and prognostic assessment for lung cancer patients [16]. This review expounds on liquid biopsy-based biomarkers and their detection methods, providing a comprehensive overview of the application of liquid biopsy in early diagnosis, treatment selection, recurrence monitoring, and prognostic evaluation in lung cancer. Additionally, the analysis extends to future trends and limitations in the hope of enhancing current management strategies for patients with lung cancer.

## Biomarkers and detection methods for liquid biopsy of lung cancer

**Fig. 1:**
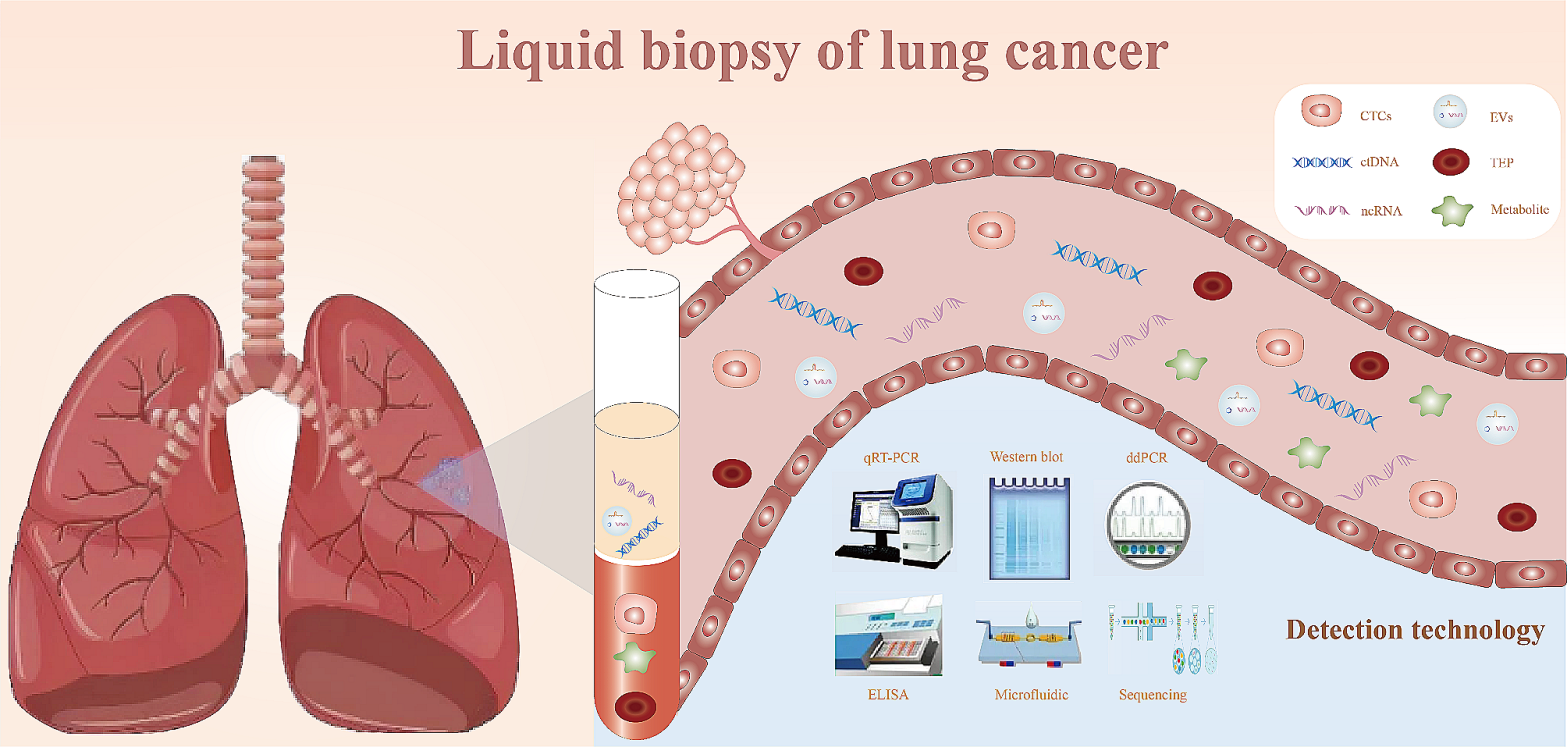
Liquid biopsy enables the early diagnosis and prognostic evaluation of lung cancer by utilizing circulating tumor cells (CTCs), circulating tumor DNA (ctDNA), non-coding RNA (ncRNA), extracellular vesicles (EVs), tumor metabolites, and tumor-educated platelets (TEPs) as biomarkers. Techniques including quantitative real-time polymerase chain reaction (qRT-PCR), western blotting, droplet digital polymerase chain reaction (ddPCR), enzyme-linked immuno sorbent assay (ELISA), microfluidics, and sequencing can be employed for the isolation and detection of liquid biopsies in lung cancer

At present, the incorporation of circulating tumor cells, circulating tumor DNA, non-coding RNA, extracellular vesicles, tumor metabolites, tumor-associated antigens, and tumor-educated platelets holds considerable potential as valuable biomarkers, steering the trajectory of liquid biopsy applications in the realm of oncology research (Fig. 1).

### Circulating tumor cells

Circulating tumor cells are cells originating from primary or metastatic lesions that enter the bloodstream either spontaneously or due to manipulations during diagnostic procedures, leading to cell shedding [17]. The presence of a significant number of metastatic precursors among CTCs heightens the risk of tumor metastasis and recurrence. It is now understood that a subset of tumor cells within the peripheral blood can evade apoptosis/phagocytosis, undergo epithelial-mesenchymal transition (EMT), and acquire enhanced mobility, adherence, invasiveness, and penetration capabilities [18, 19], ultimately promoting intravascular infiltration and resulting in distant tumor metastasis through migration in the peripheral blood [20].

Currently, blood tests for CTCs offer diverse avenues for assessing tumor-related information, including the analysis of tumor morphology or genetic information, scrutiny of tumor genotypes and heterogeneity, and effective monitoring of patient response to treatment, thereby improving clinical prognosis [21, 22]. In addition, CTCs may illuminate the distinct characteristics of tumors, guiding clinicians in providing personalized and precise treatment for patients. Substantial evidence underscores the clinical significance of CTCs in tumor progression, not only for early tumor diagnosis, metastatic recurrence, and predicting the risk of tumor progression but also for real-time monitoring of tumor drug therapy [23]. Therefore, efficient CTC detection methods can potentially facilitate early tumor detection and palliative care monitoring in patients with advanced tumors, presenting a promising outlook for the future of lung cancer treatment [24].

The primary process for elucidating tumor properties through CTCs involves enrichment, detection, and characterization of these cells [25]. Immunoenrichment and physical enrichment are the primary methods employed for CTC enrichment. Immunoenrichment can be achieved through methods such as a microfluidic chip-based immunosorbent technique and an immunomagnetic bead method [26]. The CellSearch system, as a representative immunomagnetic bead method for CTC enrichment, can automatically capture, count, and analyze stained cells, targeting epithelial cell adhesion molecules (ECAM) [27]. However, the efficacy of the CellSearch system may be impacted by the potential loss of epithelial antigens during epithelial-mesenchymal transition and variations in CTC abundance among different tumor types. Microfluidic chips form the foundation of the immunosorbent assay, utilizing various sizes and structures to manipulate blood flow patterns, thereby expanding possibilities for CTC immunosorbent techniques and antibody interactions [28]. On the other hand, physical enrichment relies on the inherent physical distinctions between CTCs and blood cells, such as size, relative density, and surface charge. Various materials and devices with different sizes are designed for the filtration and separation of CTCs [29].

Enrichment of CTCs serves the purpose of reducing background blood cell levels in peripheral blood, allowing for the detection and analysis of the remaining CTC population. Currently, diverse methods, including protein expression, immunocytochemistry, and molecular nucleic acids, are employed for the detection of CTCs [30]. Flow cytometry is commonly used to quantify and comprehensively characterize protein expression in CTCs, enabling the observation of multiple biomarker expression profiles. However, the challenge of detecting rare CTC populations remains a limitation of flow cytometry [31]. Immunohistochemical staining and immunofluorescence are widely applied for the detection and characterization of distinct CTCs [32, 33]. Immunofluorescence techniques, leveraging fluorescent labeling and isolation by specific antibodies recognizing selected markers on CTCs [33], allow for the observation of protein expression and location information. Consequently, immunofluorescence technology is a practical tool for assessing tumor protein expression through CTC detection. In recent years, numerous labeled immunofluorescence panels have been utilized to comprehensively evaluate the distribution and expression patterns of various CTC subtypes. This not only aids in determining tumor staging and subtypes but also establishes a personalized basis for the precision treatment of tumor patients [34]. Beyond these techniques, imaging histology, tumor mutation analysis, and single-cell sequencing are also employed for the detection and analysis of CTCs. These approaches contribute to mapping tumor heterogeneity comprehensively and characterizing CTCs at various histological and functional levels. Ultimately, they enable effective monitoring of the dynamic evolution of tumor heterogeneity [35].

While the exact relationship of CTCs in the process of tumor proliferation and metastasis necessitates further investigation, CTCs offer valuable information for liquid biopsy, providing a comprehensive understanding of tumor progression. In the clinical management of lung cancer patients, CTCs play an indispensable role in early diagnosis, prognostic assessment, and recurrence monitoring.

### Circulating tumor DNA

Circulating free DNA (cfDNA) consists of DNA fragments released into the bloodstream through processes such as apoptosis, necrosis, or active secretion from healthy, damaged, inflamed tissues or tumors [36]. Within cfDNA, circulating tumor DNA represents a specific subset released into the circulatory system either through shedding by tumor cells or during apoptosis, encoding the genes of the tumor cell [37]. ctDNA serves as a distinctive tumor biomarker, and its testing enables the detection of minute traces of tumors in the blood. Typically, ctDNA fragments are approximately 160–200 base pairs in length, constituting around 0.01-90% of cfDNA in peripheral blood [38]. Notably, ctDNA is less influenced by tumor heterogeneity compared to tumor tissue. Furthermore, in contrast to conventional blood proteins, which may take 2–3 weeks for markers to appear, ctDNA has a relatively short half-life, ranging from approximately 15 min to 2.5 h. This characteristic suggests that ctDNA can function as a real-time tumor biomarker, providing dynamic monitoring and reflection of tumor evolution [39]. Interestingly, ctDNA carries genetic information relevant to tumors, encompassing mutation levels, methylation status, and microsatellite instability [40]. Recently, the FDA has approved liquid biopsy-based tests for ctDNA testing in other tumor types or for validation in the clinical setting, such as the Epi proColon for colorectal cancer screening and the Signatera test for identifying molecular residual lesions and recurrence in multiple types of solid tumors [41, 42]. These advantages position ctDNA as a next-generation liquid biopsy marker superior to traditional markers. It not only aids in the early diagnosis of tumor status but also offers diverse ways to assess the prognosis of tumor patients and monitor recurrence/metastasis.

The detection of ctDNA involves critical steps such as ctDNA preparation, library construction, and data comparison and analysis. ctDNA testing predominantly focuses on gene mutations and DNA methylation, as these alterations have the potential to activate oncogenes, disrupt the balance between oncogenes and tumor suppressor genes, and stimulate tumor progression [43]. DNA methylation, in particular, can influence DNA conformation, stability, and protein interactions, thereby contributing to tumorigenesis by regulating gene expression [44]. The low abundance of ctDNA in the blood necessitates highly sensitive techniques for mutation detection. Currently, various methods such as the amplification refractory mutation system (ARMS), quantitative PCR (qPCR), digital PCR, and next-generation sequencing (NGS) enable qualitative and quantitative analysis of ctDNA with high accuracy [25]. qPCR, for instance, monitors DNA amplification in real-time with high specificity and sensitivity, making it suitable for multiplexed analysis [45]. NGS platforms offer the advantage of comprehensive detection, covering not only coding regions but also non-coding and regulatory regions, as well as structural variants. Digital PCR stands out for its high sensitivity, accurate quantification of DNA molecules, and stability in detecting rare targets amidst complex background interference [46]. Combining gene editing technologies, nanomaterials, and other integrated detection strategies with current ctDNA methods may enhance the identification of target gene mutations in ctDNA by increasing the net amount of mutant fragments [47].

In advanced or metastatic lung cancer, the use of circulating tumor DNA (ctDNA) has various applications such as predicting chemotherapy regimens and evaluating patient treatment response and survival outcomes. The detection of EGFR mutations in ctDNA serves as a highly specific molecular marker for both diagnosis and prognosis of tumors. Notably, ctDNA testing can help identify unknown tumor tissue variants, particularly in scenarios where chemotherapy leads to chemoresistance in specific tumor cells, resulting in residual clusters of cells. Therefore, ctDNA demonstrates significant utility in the early diagnosis and prognostication of lung cancer. Liquid biopsy technology is utilized to collect plasma ctDNA from NSCLC patients, enabling the detection of genetic mutations and the identification of new genetic changes associated with acquired drug resistance. Tailored treatment regimens based on these findings significantly enhance clinical care efficacy. However, akin to circulating tumor cells (CTCs), ctDNA levels in cancer patients are typically low with a short half-life, making them susceptible to interference from normal cell-free DNA (cfDNA) and necessitating highly sensitive detection methodologies. Advances in DNA sequencing technology have enabled the qualitative and quantitative analysis of ctDNA directly following DNA amplification through established detection techniques like amplification refractory mutation system (ARMS), digital polymerase chain reaction (PCR), and next-generation sequencing (NGS). In addition to standardizing the detection process, the clinical utility of ctDNA requires further validation through extensive clinical trials.

### Non-coding RNA

Non-coding RNAs (ncRNAs) are RNA molecules that do not encode proteins, including rRNA, tRNA, snRNA, snoRNA, and miRNA with known functions [48]. Despite not being involved in protein translation, they play a crucial role in regulating gene and protein expression. ncRNAs are broadly classified into small non-coding RNAs (sncRNAs, less than 200 nucleotides in length) and long non-coding RNAs (lncRNAs, more than 200 nucleotides in length) based on their length [49]. These RNAs participate in various oncogenic processes, including EMT, autophagy, and cellular senescence, by modulating gene expression, cell proliferation, and differentiation [50].

MicroRNAs, among the most extensively studied ncRNAs in oncology research, can be identified through various liquid biopsy techniques that detect miRNAs released by CTCs and tumor cells [51]. Detecting miRNAs is of paramount importance as biomarkers for the diagnostic assessment of lung cancer. For instance, miR-125, miR-21-5p, miR-200b, and miR-141 in the blood show varying correlations with lung cancer progression and tumor chemosensitivity [52–54]. Consequently, miRNAs are regarded as potential biomarkers for the early diagnosis and prognostic monitoring of lung cancer.

Long non-coding RNAs are widely thought to influence tumor progression through various life processes, including epigenetics and cell cycle regulation. Advances in sequencing technologies have facilitated the characterization of numerous lncRNAs, establishing their value as liquid biopsy biomarkers in lung cancer. lncRNAs such as H19, MIR22HG, and LINC-PINT exhibit differential expression in lung cancer tissues and are implicated in the regulation of lung cancer progression [55–57], making them potential diagnostic markers and therapeutic targets for lung cancer.

Circular RNAs (circRNAs) are covalently closed-loop molecules without a 5′ end cap structure and 3′ end poly(A) tail. Rich in miRNA binding sites, circRNAs act as miRNA sponges, relieving the inhibitory effect of miRNAs on their target genes and participating in the regulation of various diseases, including tumors [58]. Due to their closed-loop structure, circRNAs exhibit good stability, allowing for consistent and stable expression in cells, making them suitable biomarkers for early diagnosis and recurrence monitoring of lung cancer [25]. Several circRNAs, such as circFASRA, circHMGB2, and circSATB2, have been implicated in immunosuppression, chemoresistance, and tumor progression in lung cancer [59–61]. Thus, plasma circRNAs can serve not only as diagnostic markers in liquid biopsy for lung cancer but also as therapeutic targets and prognostic/recurrence monitors.

Current methods for ncRNA research, similar to those for ctDNA and CTCs, include transcriptome sequencing, real-time fluorescence quantitative PCR, fluorescence in situ hybridization, RNA interference, and immunoprecipitation of RNA-binding proteins. Among these, fluorescence quantitative PCR is widely used as the gold standard for detecting non-coding RNA expression [62]. In a study by Liang et al. [63], fluorescence signals were enhanced to improve the detection sensitivity of miRNAs and circRNAs, making them potential early diagnostic markers for tumors. Overall, these methods offer a range of options for ncRNA detection and hold great potential for the clinical screening of lung cancer.

### Extracellular vesicles

Extracellular vesicles are nanoscale phospholipid bilayer vesicles actively secreted by all cells, playing a crucial role in intercellular communication and the regulation of cellular activities. EVs carry various genetic materials, including mRNA, miRNA, lncRNA, nucleic acids, and proteins, making them essential mediators of cellular functions [64]. They actively participate in tumorigenesis, progression, and the regulation of the immune microenvironment through cellular communication under specific physiological and pathological conditions, owing to their penetrating and migratory abilities [65]. Based on the recommendations of the Extracellular Vesicle Association, EVs of different origins and sizes are classified into three types: exosomes (30–150 nm), microvesicles (200-1,000 nm), and apoptotic vesicles (800-5,000 nm) [65]. Exosomes, in particular, are the most extensively studied subtype of EVs due to their stability and penetration ability, influencing the life progression of tumor cells through immune regulation, signaling, and molecular transport [66]. Crucially, EVs released by living cells and the materials they carry provide a more accurate real-time reflection of tumor foci than the small amounts of circulating tumor DNA and possibly necrotic circulating tumor cells in peripheral blood [67]. The stability of EVs and their ability to characterize tumor status make them a promising alternative tool to liquid biopsy, offering new options for the diagnosis and prognosis of lung cancer.

In recent years, it has become increasingly evident that genetic information, such as miRNAs and lncRNAs loaded in extracellular vesicles, plays a crucial role in the regulation of tumor progression through cellular communication. These genetic elements have emerged as promising biomarkers with excellent prospects. For instance, miRNAs like miR-934, miR-186-5p, miR-497-5p, and miR-29a-3p carried by EVs have been identified as regulators of lung cancer progression through different signaling axes, positioning them as potential biomarkers [68–70]. Similarly, EVs-derived lncRNAs such as ZEB2-AS1, UFC1, and circUSP7, along with circSATB2 and tumor-derived exosomal proteins, have demonstrated potential for the early diagnosis of lung cancer [59, 71–74]. Therefore, unraveling the genetic content of EVs, including miRNAs, circRNAs, and lncRNAs, may be pivotal in understanding and diagnosing lung cancer.

The development of extracellular vesicles in liquid biopsies faces numerous challenges, primarily the low abundance of EVs in biological samples, which poses limitations on their isolation and characterization. Various techniques are currently employed for the separation of EVs, including ultracentrifugation, ultrafiltration, molecular exclusion chromatography, polymer precipitation, immunoaffinity chromatography, and microfluidics [64]. To enhance the efficiency of EVs separation, a combination of multiple techniques is often necessary, considering factors such as experimental conditions, procedural contamination, and sample characteristics [64]. Each separation technique has its advantages and disadvantages, such as the time-consuming nature of ultracentrifugation, susceptibility to contamination and denaturation of ultrafiltration, and the low content of immunoaffinity chromatographic purification [75, 76]. Notably, microfluidics stands out for its portability, rapidity, low cost, ease of handling, and low contamination rate compared to other EVs separation techniques, making it a promising option for EVs separation and purification. A novel microfluidic technique utilizing zinc oxide nanowires and surface charge has been developed to enhance EVs capture efficiency [77]. However, these separation methods require further refinement to optimize EVs separation efficiency and advance their clinical applications, especially in areas such as early tumor screening and prognostic evaluation [78].

Following the purification of EVs, their identification and characterization are essential for detecting potential biomarkers they carry. Exosomes, for instance, are commonly identified through EV tracing, transmission electron microscopy, and particle size detection [79]. Moreover, immunoblotting, enzyme-linked immunosorbent assay (ELISA), and flow cytometry are frequently employed to detect proteins carried by EVs [80]. The continuous development of new technologies, such as electrochemistry, colorimetry, and nano-biosensors, provides additional possibilities for the identification and characterization of EVs, enhancing the sensitivity of EVs detection [81]. Despite the promising biological properties of EVs for the diagnosis, prognosis, and treatment of lung cancer, there is an urgent need to optimize isolation techniques and improve the clinical translational efficiency of relevant EVs.

### Tumor-educated platelet

During tumor tissue development, tumor cells can influence the RNA message and protein levels of platelets through various signaling molecules or receptors, leading to the formation of tumor-educated platelets (TEPs). TEPs provide abundant spliced RNA biomarkers and RNA profiles that hold potential for tumor detection [82, 83]. Numerous studies have verified that the number and size of TEPs can reveal clinically relevant information related to tumors, such as tumor presence, location details, and molecular signatures. Moreover, these factors are associated with increased mortality in several cancers, including lung cancer [84, 85]. For instance, tumor-derived platelet factor 4 (PF4, CXCL4) has been shown to promote bone marrow megakaryocyte-mediated thrombopoiesis in NSCLC patients, and circulating platelets can contribute to tumor progression by modulating the tumor immune response [86, 87]. A recent study suggested that platelets in the peripheral blood of NSCLC patients could help characterize clinically relevant biomarkers and provide information about the spread and metastasis of tumor foci [88].

The process of detecting platelet RNA involves platelet isolation and sequencing of RNA transcripts. Various techniques, including platelet RNA sequencing, microarray hybridization techniques, and reverse transcriptase polymerase chain reaction (qRT-PCR), have enabled platelet RNA to fulfill its significant oncological diagnostic potential in blood liquid biopsies for lung cancer [89–91]. Additionally, RNA sequencing in next-generation sequencing has gained attention for its ability to resolve multiple genetic information simultaneously [92]. It is important to note that complex isolation procedures and the resolution of tumor markers from platelet RNA pose challenges. Currently, a critical issue to address is avoiding interference and contamination of TEPs by other biomarkers (e.g., ctDNA and EVs) during the isolation of TEPs and the resolution of tumor-associated markers [93, 94]. In preclinical studies, there is an urgent need to optimize and simplify the platelet extraction and RNA sequencing process to reduce time consumption and expense costs.

### Metabolites

Tumor growth and progression often induce physiological changes in the body, particularly alterations in overall metabolic status. Tumor cells modify their metabolic pathways during proliferation and metastasis, releasing metabolites into the bloodstream. These metabolites can serve as tumor markers in liquid biopsies for the early diagnosis, prognostic evaluation, therapy response and recurrence monitoring of lung cancer [95].

Crucially, glucose metabolism plays a pivotal role in tumor cell proliferation, with glucose metabolites such as 2-hydroxyglutaric acid, succinic acid, and fumaric acid [96] being significant signaling molecules. Growing evidence suggests that these metabolites may regulate tumor progression through epigenetic enzymes and DNA repair, and they have shown associations with survival and clinical prognosis in cancer patients [96, 97]. Lactate, a major glycolysis metabolite, contributes to tumor metastasis through histone modifications that regulate gene expression and reprogram the tumor microenvironment [98, 99]. Additionally, products of tumor lipid and amino acid metabolism offer diagnostic possibilities. For instance, plasma lipid metabolites like arachidonic acid and linoleic acid are identified as potential clinical diagnostic markers regulating lung cancer progression through the Akt pathway [100]. Amino acid metabolites, including tryptophan, leucine, and valine, serve as valuable biomarkers to differentiate between tumor and non-tumor patients [101, 102].

Metabolomics technologies, relying on nuclear magnetic resonance (NMR) and mass spectrometry (MS), are instrumental in the comprehensive detection and accurate quantification of tumor metabolites [103]. NMR, as a spectroscopic technique, is increasingly employed for metabolic fingerprint research and in vivo studies [104]. In addition, MS is typically classified into three types: capillary electrophoresis-mass spectrometry (CE-MS), gas chromatography-mass spectrometry (GC-MS), and liquid chromatography-mass spectrometry (LC-MS) [105]. MS is often coupled with chromatographic separations to enhance the efficiency of extensive analysis and accurate quantification of metabolites. However, the challenges posed by the low levels of metabolites in peripheral blood and the complexity of sample preparation for metabolomics have driven technological advances. For instance, ion mobility spectrometry coupled to mass spectrometry (IMS-MS) has been developed to avoid detection overlap and extend the range of detectable metabolites. The strategy of acquiring MS1 and MS2 spectra simultaneously in IMS-MS improves the accuracy of identifying metabolic biomarkers [106, 107]. Additionally, different assay strategies vary, and a combination of multiple strategies may be advantageous. Simultaneous acquisition of MS1 and MS2 spectra improves the accuracy of identifying metabolic biomarkers. The combined use of non-targeted metabolomics based on the data-dependent acquisition (DDA) model and targeted metabolomics based on the multiple reaction monitoring (MRM) model enhances sensitivity for metabolite detection [106, 108].

The intricate and dynamic evolution of metabolites presents a challenge to the application of metabolomics. To overcome this challenge, there is a need for improved metabolomics methods and instrumentation. These advancements should aim to address the current limitations, including limited metabolite detection coverage, insufficient detection sensitivity, low qualitative and quantitative accuracy, and the absence of spatial information about the tumor [109].

### Tumor-associated antigens

Tumor-associated antigens (TAAs) are molecular markers found on both tumor cells and normal cells, encompassing various types like embryonic proteins, glycoprotein antigens, and squamous cell antigens. Although TAAs are not exclusive to tumor cells, they are produced in trace amounts by normal cells and notably highly expressed actively dividing tumor cells, earning them the term “associated antigens.” Recent investigations indicate that the abnormal expression of TAAs in the bloodstream may contain valuable information about tumor cell activity, size, gene mutations, and more. In a study conducted by Li and colleagues [110], the presence of autoantibodies in the serum of patients with lung cancer was identified by enzyme-linked immunosorbent assay (ELISA). The study suggested that increased expression of serum anti-cytosine B1, anti-sufflatoxin autoantibodies, and serum anti-p53 autoantibodies might indicate tumor proliferation.

Consequently, these biomarkers have promising potential for non-invasive early diagnosis of lung cancer. Additionally, the dysregulation or increased expression of TAAs is closely associated with genetic mutations. Notably, the well-studied TAA, p53, when deleted or mutated in tumors, affects myeloid and T-cell recruitment and activity. This phenomenon the tumor to evade the immune system and promotes cancer growth. Additionally, the role of p53 in immune cells can lead to various effects that may impede the progression of the tumor [111]. Moreover, other well-known TAAs, such as cancer-testis antigens (for instance, GAGE1 and NY-ESO-1) and oncofetal proteins, play a crucial role in the development of cancer. These TAAs offer promising avenues for the early diagnosis of NSCLC [112].

Due to the inherent challenges in identifying TAA in lung cancer patients, current research predominantly relies on the recognition of known TAAs, coupled with the detection of corresponding autoantibodies through immunoreactivity in patient sera. Primary techniques employed for TAA identification include serological analysis of recombination cDNA expression libraries (SEREX) and protein microarrays. SEREX, a classical experimental method, involves screening patient sera against cDNA expression libraries to autoantibodies through serological analysis [113]. This unbiased approach to transfer membranes systematically screens TAA autoantibodies (TAAb) [114]. For instance, Li and colleagues managed to isolate 57 TAAs from a T7 phage library specific to NSCLC. Utilizing a combination of TAAb with five antigens (HSP70, HSP90, p130, GAGE, and BMI-1) resulted in significant diagnostic accuracy, evidenced by an area under the curve (AUC) of 0.840, a sensitivity rate of 82.0%, and a specificity rate of 83.0%. This highlights the method’s effectiveness in pinpointing potential markers for lung cancer using TAA [115]. Protein microarrays served as practical tools for detecting TAAs in cancer patients. This process entails the deposition of purified recombinant proteins onto a chip using microarray printing methods, which is then probed with the patient’s serum. This is followed by incubation with specific antibodies to detect reactive antigens. Screening for antigens using protein arrays allows for the monitoring of an organism’s proteome, facilitated by the use of comprehensive separation methods [116]. For example, Zhong and colleagues [117] pinpointed GAGE7, EEF1A, PMS2P7, NOLC1, and SEC15L2 utilizing a protein microarray that comprised 212 immunoreactive phages, which are significantly linked to different signaling pathways in lung cancer. Similarly, Shan et al. [112] identified five TAAs (XAGE-1, ADAM29, NY-ESO-1, MAGEC1, and p53) using a microarray containing 72 proteins, confirming their connection to the progression of lung cancer through ELISA.

## Clinical application potential of liquid biopsy in lung cancer

**Fig. 2:**
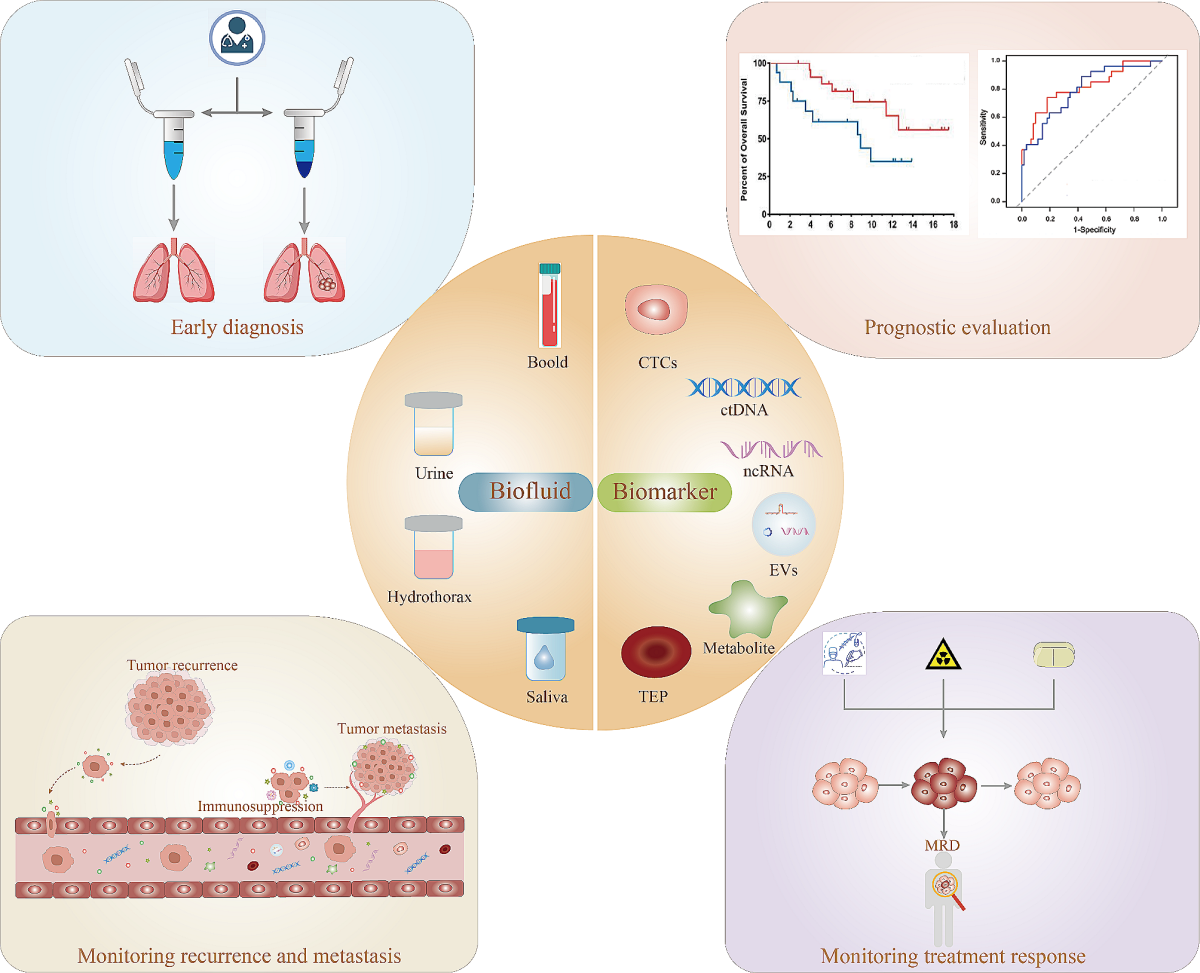
Clinical potential of biomarkers (CTCs, ctDNA, ncRNA, EVs, tumor metabolites, TEP) in different biofluids (blood, urine, pleural fluid, saliva) for early diagnosis, prognostic evaluation, monitoring treatment response, recurrence and metastasis monitoring of lung cancer

In recent years, liquid biopsy has been seamlessly integrated into clinical practice, as illustrated in Fig. 2. The identification of multiple tumor biomarkers in body fluids has elevated liquid biopsy to a pivotal role in the early screening and diagnosis of lung cancer, as well as in monitoring treatment responses and assessing the prognosis of tumor recurrence and metastasis (Table 1).

**Table 1:** The clinical potential of liquid biopsy in the diagnosis, treatment monitoring, and prognostic evaluation of lung cancer

	Biomarker	Type	Findings	Ref
Disease diagnosis	CTCs	CTCs	Increased expression levels of CTCs or CA125 help in diagnosis of NSCLC	[245]
		CTCs	Combination of CTCs with the lung cancer biomarker CEA improves early diagnosis of lung cancer	[246]
		PD-L1	The detection of CTCs and PD-L1 in peripheral blood is helpful for the diagnosis of lung cancer patients.	[247]
	ctDNA	DNA methylation	DNA methylation sequencing of ctDNA allows non-invasive diagnosis of early lung cancer	[4]
		ctDNA sequencing	ctDNA helps detect early lung cancer	[248]
		Tumor mutation	Tumor mutation ctDNA analysis is something that can help with early lung cancer diagnosis.	[249]
	ncRNA	miR-155-5p	Early diagnosis of lung cancer via miR-155-5p	[250]
		LINC00313/miR-4429	LINC00313/miR-4429 informs NSCLC diagnosis and prognosis	[251]
		miR-205-5p	miR-205-5p promotes lung cancer progression and is valuable in the diagnosis of lung cancer	[252]
		miRNA-21	miRNA-21 can be used as a biomarker for early diagnosis of lung cancer	[253]
	EVs	miR-21, miR-191	Extracellular vesicles isolated and detected in plasma can be used for lung cancer diagnosis	[254]
		NG	Detection of extracellular vesicular membrane proteins by Förster resonance energy transfer for accurate and convenient early diagnosis of lung cancer	[255]
		miR-520c-3p/miR-1274b	miR-520c-3p and miR-1274b in EVs of lung cancer patients contribute to diagnosis of NSCLC	[139]
	Metabolites	Cytosine, lysine, tyrosine	Salivary diethanolamine, cytosine, lysine and tyrosine help in the differential diagnosis of lung cancer and benign lung lesions	[256]
		Ornithine	Tumor metabolites such as ornithine have potential for early lung cancer screening	[141]
	TEP	lncRNA	lncRNA isolated from TEP enables lung cancer diagnosis and progression prediction	[220]
		SNORD55	SNORD55 in TEP could be a potential biomarker for early diagnosis of NSCLC	[146]
	TAA	NRP2	High expression of NRP2 facilitates early diagnosis of lung cancer	[149]
		HMGB3	Autoantibody against HMGB3 has the potential to serve as a serological biomarker in early-stage lung cancer	[150]
Treatment monitoring	CTCs	CTCs	Monitoring drug resistance mutations in lung cancer patients using CTCs to assess treatment response	[257]
		CTCs	CTCs can dynamically monitor the therapeutic effect of pembrolizumab in metastatic NSCLC	[258]
	ctDNA	ctDNA sequencing	ctDNA analysis can identify residual/recurrent disease earlier than standard radiography, thus contributing to early personalized adjuvant therapy	[259]
		ctDNA mutation	Evaluation of ctDNA predicts tumor recurrence in lung cancer	[260]
		ctDNA mutation	ctDNA can monitor for microscopic residual disease	[261]
		ctDNA sequencing	Detection of microscopic residual disease after lung cancer surgery	[262]
	ncRNA	microRNA	microRNAs can be used as biomarkers in NSCLC to monitor therapeutic response to chemotherapy in tumor cells	[263]
		circ_0011292	circ_0011292 is associated with NSCLC progression and chemotherapy resistance	[264]
	EVs	miRNA-30c	Extracellular vesicular miRNA-30c monitors response to radiotherapy treatment and assesses prognosis in lung cancer patients	[265]
		circ_0041150	Monitoring chemoresistance in NSCLC treated with chemotherapy regimens by circ_0041150 in lung cancer cell-derived EVs	[266]
	Metabolites	SN-38	Monitoring the therapeutic response of irinotecan in tumor patients by active metabolite (SN-38)	[267]
			Tumor-derived metabolomics data can assess disease staging and chemotherapy response in NSCLC	[268]
	TEP	Tumor-derived RNA	TEP can help liquid biopsy monitor treatment resistance	[93]
		PD-L1	PD-L1 expressed on TEP can influence immune checkpoint therapy response	[269]
	TAA	TRIM21	Positive TRIM21 autoantibody combinations in peripheral blood of lung cancer are significantly associated with better treatment response	[186]
		HMGB1	HMGB1 may predict treatment response and prognosis in patients with advanced NSCLC	[188]
Prognosis evaluation	CTCs	CTCs	Circulating tumor cells in patients with lung cancer universally indicate poor prognosis	[270]
		DNA Methylation	DNA methylation sequencing of CTCs predicts prognosis and provides therapeutic strategies for lung cancer	[21]
	ctDNA	ctDNA	ctDNA predicts prognostic outcomes and tracks early tumor dissemination in lung cancer patients	[271]
		ctDNA	Combined circulating tumor DNA and protein biomarker assay analyzes prognosis of lung cancer patients	[272]
	ncRNAs	lncRNA	Immune checkpoint-associated lncRNA identify different subtypes of lung cancer and predict immunotherapy and prognosis	[273]
		lncRNA AC079630.4	Expression of lncRNA AC079630.4 is associated with poor prognosis in lung cancer patients	[274]
	EVs	hY4 RNA	EVs carrying hY4 RNA methylation fragments promote lung cancer progression	[275]
		miR-126/miR-320	miR-126 and miR-320 in EVs of lung cancer origin induce lung cancer metastasis and are associated with poor prognosis	[276]
	Metabolites	Creatine/creatinine	Tumor metabolites (creatine and creatinine) shown to be useful in assessing lung cancer prognosis and monitoring cancer recurrence	[277]
		Creatine nucleoside	Tumor metabolites creatine nucleoside and arginine as potential indicators of poor prognosis in lung cancer	[278]
	TEP	Volume and number of TEP	Volume and number of TEP can inform the evaluation of lung cancer prognosis	[279]
		ITGA2B	ITGA2B in TEP can be applied to diagnostic and prognostic evaluation of NSCLC	[148]
	TAA	MAGE-B2	MAGE-B2 in lung cancer serum can reflect tumor recurrence and metastasis information	[223]

NG: not given

### Lung cancer diagnosis

The current methods for detecting and diagnosing lung cancer, such as EUS-FNA, MRI, and CT [118], rely on invasive procedures that may pose risks to patients. While pathological tissue testing remains the gold standard, it is essential to acknowledge the invasiveness of this approach. Given the substantial time gap between the formation of lung cancer and the manifestation of symptoms spanning several years, liquid biopsy emerges as a crucial tool for early screening. This presents a valuable opportunity to enhance treatment outcomes and survival rates for lung cancer patients [119].

CTCs can be present at all stages of lung cancer progression, yet characterizing their status in the blood becomes more feasible at advanced stages due to the crucial role of CTCs dissemination in distant metastasis [120]. The limited presence of circulating tumor cells in the bloodstream during the early stages of tumor initiation presents a significant challenge for the prompt diagnosis of lung cancer. Fortunately, recent technological advancements have made substantial strides in addressing these challenges associated with CTC applications [121]. Notably, the implementation of CellCollector® in vivo CTC capture technology and CTCs next-generation sequencing has demonstrated a commendable CTC detection rate of 62.5% for type I/II NSCLC [121, 122]. In addition to these advancements, the identification of four high-frequency mutated genes (NOTCH1, IGF2, EGFR, and PTCH1) through CTCs NGS holds promise for the early diagnosis of lung cancer [123]. Furthermore, the application of NGS analysis to CTCs has unveiled dozens of differentially expressed metabolites, with 10 specific metabolites showing potential clinical value in diagnosing early-stage lung cancer [124]. The distinctive scarcity of CTCs in the blood of non-tumor individuals underscores their specificity as biomarkers for early lung cancer diagnosis. However, it is crucial to acknowledge the pressing need for ongoing development and optimization of additional techniques to enhance the sensitivity of CTC detection.

Besides, circulating tumor DNA presents a compelling potential for early-stage lung cancer diagnosis, originating from apoptotic or necrotic tumor cells. Significantly elevated plasma ctDNA levels in lung cancer patients, compared to patients with respiratory inflammation, underscore its diagnostic relevance. With a sensitivity of 90% and specificity of 80.5%, ctDNA proves effective in differentiating between lung cancer patients and healthy individuals [125]. Notably, a study revealed ctDNA detection in 50% of plasma specimens from stage I NSCLC patients, reaching 100% as the tumor progressed to stages II-IV [126]. The correlation between ctDNA levels and tumor volume, along with its ability to distinguish residual foci from treatment-related imaging changes, highlights its potential for earlier tumor assessment compared to traditional imaging methods [127]. It is essential, however, to approach the analysis of ctDNA mutations comprehensively, considering the possibility of their association with non-neoplastic cells and potential implications for early tumorigenesis [128]. Hence, it is crucial to approach the significance of these mutations in ctDNA analysis with a comprehensive and cautious perspective to assess the potential for early tumorigenesis. Previous studies have successfully utilized blood ctDNA to determine the mutational status of the epidermal growth factor receptor (EGFR) and establish connections between EGFR mutations in ctDNA and the progression status of lung cancer. An in-depth analysis of patients with EGFR mutations in ctDNA indicated that the L858R mutation, whether identified in tumor tissue or ctDNA, serves as a marker for shortened overall survival (OS) and progression-free survival (PFS), which underscores the potential of the L858R mutation in ctDNA as both a diagnostic and prognostic marker for lung cancer [129]. Notably, the methylation status ctDNA holds promise for contributing to the early diagnosis of lung cancer. Although further clinical trials are necessary for optimization, methylation markers present a compelling and noninvasive diagnostic strategy for ctDNA in the early diagnosis of lung cancer [4, 119].

Definitive studies have underscored the promising biomarker potential of miRNAs due to their extensive involvement in regulating multiple mechanisms of lung cancer proliferation and invasion. Despite this, there remains a gap in the widespread use of miRNA in the clinical diagnosis of lung cancer. Notably, miR-1246 and miR-1290 are implicated in correlating with tumor stage and clinical response in NSCLC, as evidenced by their significantly elevated expression levels in the blood of tumor patients compared to the healthy population [130]. An intriguing report sequencing NSCLC tissue samples has suggested that specific LCS6 allele variants could serve as potential risk factors for NSCLC [131]. Consequently, detecting information related to miRNAs in the body fluids of NSCLC patients holds promise as a potential noninvasive method for early lung cancer diagnosis. Notably, Abdollahi et al. [132] have proposed a combined miRNA diagnostic method for NSCLC, presenting a miR-panel (miR-21, miR-148, miR-152, and miR-638) with a sensitivity and specificity of 86.67%. This finding further advocates for the clinical application of miRNA in the early diagnosis of lung cancer. However, it is crucial to emphasize the efficacy of individual miRNAs in lung cancer diagnosis to strike a balance between effectiveness and cost. While specific diagnostic models containing multiple biomarkers exhibit good predictive diagnostic efficacy, their low reproducibility and complex composition pose substantial challenges for the practical application of miRNAs in the early diagnosis of lung cancer [133]. Advancements in sequencing technology have led to the identification of various lncRNAs and circRNAs that exhibit differential expression in lung cancer blood and tissue. There is growing evidence that the expression of specific lncRNAs and circRNAs may offer valuable insights into the clinical features of lung cancer [134]. For instance, circFASRA and lncRNA PCAT1 have emerged as potential diagnostic markers for lung cancer [135, 136]. Currently, the analysis of lncRNA and circRNA relies predominantly on bioinformatics data prediction, highlighting the need for additional experimental and clinical studies to substantiate and confirm their diagnostic value in the future.

EVs carry a diverse range of biological information, and the characterization of tumor cell-derived EVs has gained significant momentum as potential biomarkers for the early diagnosis of lung cancer [137]. A study by Yuan et al. [138] utilizing microfluidics to analyze EVs in plasma revealed their ability to recognize lung cancer-related markers PTX3 and THBS1, along with the typical EV marker CD63. Employing a machine learning-based EVs nano-strategy, the study achieved an impressive 92.3% sensitivity and 100% specificity in distinguishing early-stage lung cancer from benign lung disease, outperforming CT scan-based lung cancer diagnosis (92.3% sensitivity and 71.4% specificity). This suggests that EVs can serve as biomarkers for the detection of precancerous lesions. Current methods for diagnosing early lung cancer using EVs are inclined towards the detection of miRNA or lncRNA carried by EVs [139, 140]. For example, the elevated expression of miR-520c-3p and miR-1274b in lung cancer can be detected by EVs for the early diagnosis of NSCLC [139]. However, the standardization of techniques for the isolation and detection of EVs is essential to facilitate the full implementation of EVs in the early diagnosis of lung cancer, and broader clinical studies are indispensable for further validation.

The use of high-resolution mass spectrometry is increasingly prevalent for detecting tumor metabolites as potential biomarkers for the early diagnosis of tumors. Metabolomics enables the analysis of metabolites released into the bloodstream due to the metabolic reprogramming of tumor tissues [141]. A study confirmed that specific metabolites detected in plasma, such as palmitic acid, heptadecanoic acid, 4-oxoproline, and tridecanoic acid, demonstrate discriminatory accuracies and area under the curve (AUC) scores of up to 0.829 and 0.869 for early-stage lung cancer, highlighting the potential of tumor metabolites as diagnostic biomarkers [141]. Additionally, Guan et al. developed a prediction model for early lung cancer based on metabolic indicators and identified metabolic biomarkers like ornithine and palmitoyl carnitine as potential candidates for screening lung cancer (AUC = 0.81, accuracy = 75.29%, sensitivity = 74%) [142]. Notably, valine, leucine, and isoleucine may also serve as potential early lung cancer diagnostic biomarkers [143]. However, the heterogeneity of different tumor subtypes may entail different metabolic modes and types of metabolites, necessitating future studies to explore more sensitive and specific markers for the early identification of different subtypes of lung cancers to facilitate the effective selection of clinical protocols [144].

In the early stages of tumorigenesis, both RNA and proteins of TEPs are changed in tumor patients, and detection of this molecular information, which is differentially expressed in healthy individuals/tumor patients, offers a potential means for early diagnosis of lung cancer [145]. For example, small nucleolar RNA (snoRNA) levels were found to differ significantly between NSCLC and normal tissues. Specifically, SNORD55 exhibited significant reduction and demonstrated good diagnostic performance for NSCLC (area under the curve, AUC of 0.803) in tumor-educated platelets of NSCLC patients [146]. Additionally, TEPs enriched with abundant circRNA is advantageous for the diagnosis of NSCLC. Various circRNAs were found to be significantly differentially expressed between asymptomatic individuals and NSCLC patients. Both reverse transcription-quantitative polymerase chain reaction (RT-qPCR) and RNA sequencing results indicated that circNRIP1 was notably downregulated in the TEPs of NSCLC patients [147]. Furthermore, TEP-derived ITGA2B has been proposed as a promising marker for improving the identification of stage I NSCLC patients and differentiating between malignant and benign lung nodules. In summary, TEPs hold the potential to serve as early diagnostic markers for NSCLC [148].

The identification of unusual alterations in TAAs within a patient’s serum presents a promising avenue for the early diagnosis of lung cancer as tumor growth takes place. Numerous crucial TAAs associated with lung cancer have been identified, participating in various biological processes during the onset and progression of the disease. Yuan and colleagues discovered several antigens, such as EEF1G, RPS3, NKAP, B2M, HSP90AB1, RAB10, EIF3D, and NRP2, associated with lung cancer. Their study revealed that a combination of eight corresponding TAAbs enabled early diagnosis with remarkable accuracy. The profile achieved an impressive AUC of 0.969, demonstrating a validity of 90.8% and a specificity of 94.1% in differentiating lung cancer patients [149]. Additionally, serum analysis revealed the sensitivity of autoantibodies to five TAAs (HMGB3, ZWINT, GREM1, NUSAP1, and MMP12) at 57.1%, 42.4%, 38.0%, 36.4%, and 20.7%, respectively. With areas under the ROC curve (AUC) of 0.85, 0.75, 0.71, 0.73, and 0.70, this highlights the substantial diagnostic potential of autoantibodies in identifying lung cancer [150]. However, it is important to recognize that not every cellular antigen detected by autoantibodies in the serum of cancer patients qualifies as a TAA. The presence of antibodies against self-proteins in the serum of cancer patients does not necessarily imply these proteins play a role in cancer development. Consequently, additional research is required to accurately identify genuine TAAs and distinguish them from antigens that might also appear in non-cancer patients and healthy individuals. Such efforts are directed towards enhancing the specificity of early lung cancer diagnosis [151].

### Monitoring treatment response

Surgical resection remains the most effective treatment for early-stage lung cancer. However, for patients with advanced lung cancer who are ineligible for surgical resection, chemotherapy becomes a crucial option to alleviate clinical symptoms and extend survival time [152]. Despite its efficacy, chemotherapy resistance poses a significant challenge, prompting the exploration of strategies such as liquid biopsy to evaluate treatment response and develop personalized regimens [153]. Several sequencing technologies now enable the characterization of molecular features in CTCs, offering insights into the treatment response of cancer patients. Sequencing technologies for CTCs provide a means to monitor chemotherapy resistance by analyzing the genes they carry, including mutations in KRAS, HER2, and TP53 [154, 155]. Additionally, assessing the programmed death-ligand 1 (PD-L1) status of CTCs enables assessment of the efficacy of monoclonal antibody treatment in lung cancer patients [156]. Notably, analyzing the expression of the chemokine receptor CXCR4 on CTCs can help identify patients likely to benefit from or develop resistance to lung cancer therapy [157, 158]. For survival outcomes in the context of chemotherapy resistance and radiotherapy, the persistence of CTCs during treatment may indicate a poor prognosis and resistance to chemotherapy in advanced NSCLC [159]. Furthermore, the number, subtype, and human telomerase reverse transcriptase (hTERT)-positive expression of CTCs in lung cancer patients are strongly associated with survival outcomes in patients undergoing radiotherapy [160]. In summary, the detection and analysis of CTCs prove valuable for evaluating and monitoring patient response to treatment, offering insights that may contribute to future clinical decisions.

The comprehensive resolution of ctDNA enables dynamic monitoring of disease trajectories, providing insights into patient responses to therapy and the emergence of microscopic residual lesions. It can be considered a valuable biomarker for tracking lung cancer progression [161]. Prior studies have demonstrated the utility of ctDNA in monitoring disease progression, especially in patients treated with tyrosine kinase inhibitors (TKIs). By identifying mechanisms of chemotherapy resistance, ctDNA allows for the adjustment of clinical treatment strategies [162, 163]. In cases where EGFR TKIs are utilized, it is crucial to check for the most common resistance mutation on EGFR, T790M. Detection of T790M in ctDNA allows for the adjustment of the dosing regimen of Osimertinib [164, 165]. If T790M is not detected in ctDNA, a tissue biopsy is necessary to confirm results and rule out false negatives from blood ctDNA testing. Additionally, it helps rule out other mechanisms of resistance, such as small-cell transformation [166]. Notably, ctDNA is believed to detect minimal residual disease (MRD) before it becomes apparent through standard clinical examination [167]. Studies have confirmed that ctDNA testing can detect MRD earlier, on average, by 5 months compared to radiography or other clinical methods [167]. Another study focused on perioperative ctDNA for detecting MRD markers in patients with stage I-III non-small cell lung cancer (NSCLC). The results indicated that perioperative ctDNA was more relevant in predicting recurrence-free survival than other factors, including tumor TNM stage [168]. While the use of ctDNA monitoring in lung cancer cases is still evolving, these studies underscore the potential of ctDNA as a biomarker for the dynamic monitoring of patients’ responses to treatment and MRDs.

Therapeutic resistance poses a significant challenge for tumor patients undergoing conservative treatment, and specific ncRNAs are implicated in the development of treatment resistance in lung cancer. For instance, miR-30b and miR-30c can predict the response to tyrosine kinase inhibitors in NSCLC, while miRNA-20a promotes chemoresistance in NSCLC through the PTEN/PI3K-AKT pathway [169, 170]. The expression of miR-16, miR-29a, miR-144, and miR-150 in blood can be indicative of tumor response to radiation therapy and patient prognosis [171]. These findings suggest the potential of miRNA levels as informative indicators of disease progression for dynamically monitoring and assessing treatment responses in tumor patients. Accordingly, Shen et al. [172] constructed a novel m6A-associated lncRNA model for predicting prognosis and response to chemotherapy and immunotherapy in lung adenocarcinoma patients to identify lung cancer patients suitable for immunotherapy and to predict sensitivity to chemotherapeutic agents. In addition, circRNA_0006420 was suggested to exacerbate lung cancer radiotherapy resistance through HUR/PTBP1 [173]. These findings highlight the potential of ncRNAs as a valuable tool for monitoring lung cancer treatment response and assessing tumor drug resistance.

EVs play a crucial role in acquired drug resistance, particularly with prolonged exposure to chemotherapeutic agents. Studies have demonstrated that the addition of cisplatin to lung cancer cells (A549 cells) promotes the secretion of EVs, contributing to the development of chemoresistance in lung cancer cells [174]. Notably, significant changes in the expression levels of several miRNAs and mRNAs associated with cisplatin sensitivity were observed in EVs. These changes mediated cisplatin resistance in A549 cells, emphasizing the potential of monitoring the formation and release of EVs to assess therapeutic responses in lung cancer cells [175]. However, it is essential to note that the development of tumor drug resistance is not always irreversible. Macitentan has been shown to reverse tumor resistance and improve tumor immunotherapy by inhibiting tumor cell-derived EVs [176]. These results suggest that EVs can serve as valuable tools for monitoring treatment responses in lung cancer patients and for developing targeted oncological strategies involving EVs.

It is now understood that metabolic reprogramming plays a significant role in tumor progression and induces chemoresistance in tumor cells to support their further development [177, 178]. For instance, glucose metabolism in lung cancer cells contributes to resistance against the antitumor activity of cisplatin [177]. The completion of tumor cell metabolism releases metabolites into the tissues or bloodstream, making the detection of relevant metabolites a promising option for monitoring responses to therapy in lung cancer patients. In a recent study, a novel in vitro cellular immune response was developed for the detection of lung cancer by evaluating glycolytic metabolic pathways and metabolites to analyze the level of immune cell activation and associated metabolite biomarkers [179]. Additionally, Li et al. [180] conducted a liquid biopsy-based metabolic phenotyping of single cells from lung cancer patients, revealing extensive metabolic heterogeneity of tumor cells. They predicted the killing effect of the drug-resistant pilot receptor tyrosine kinase AXL on tumor cells through glycolytic phenotypes and metabolites. Although metabolites have demonstrated good therapeutic response surveillance, the complex crosstalk of metabolic programming needs to be resolved before their clinical application. Glycemic metabolites do not always imply chemoresistance, and products released by other metabolic modalities (lipid metabolism, amino acid metabolism) are of equal concern [181, 182].

TEPs not only serve as biomarkers for lung cancer diagnosis but also have the potential to monitor the response to tumor therapy [183]. Studies suggest that platelets from lung cancer patients can carry fusion transcripts from tumor cells, and monitoring EML4-ALK rearrangement fusion transcripts can predict the therapeutic response to ALK inhibitors in lung cancer patients [88]. RT-PCR, with 65% sensitivity and 100% specificity for the detection of EML4-ALK rearrangements in platelets, demonstrates the noninvasive detection of these rearrangements. As patients with ALK rearrangement NSCLC often relapse within a year of treatment due to acquired resistance, it is crucial to use TEPs to identify resistance mutations to crizotinib in NSCLC [184]. Liu et al. [185] suggested that platelet mRNA groups (MAX, MTURN, and HLA-B) are associated with chemotherapy outcomes, and high expression of MAX, MTURN, and HLA-B may be linked to poor chemotherapy outcomes in lung cancer patients. These studies highlight the vital role of TEPs in the therapeutic monitoring of lung cancer, serving as a liquid biopsy marker for the disease.

Advances in oncology have significantly enhanced our understanding of the role of TAA in the response to tumor therapy, providing an emerging target for personalized tumor immunotherapy and chemotherapy. Additionally, TAAs serve as potential predictors of tumor survival prognosis and the effectiveness of immune checkpoint blockade [186, 187]. In a study focusing on NSCLC, analyzing five TAAs (p53, BRCA2, HUD, TRIM21, and NY-ESO-1) in the peripheral blood of lung cancer patients demonstrated a significant positive correlation in the combination of autoantibodies against these TAAs. This correlation was associated with a better objective response rate (ORR: 44.4% versus 13.6%, *P* < 0.001) and improved progression-free survival (PFS) (7.6 m versus 3.3 m, *P* < 0.001). These results suggest that autoantibodies against these TAAs could serve as potential predictive biomarkers for evaluating response and toxicity in treatments involving immune checkpoint inhibitors for advanced NSCLC [186]. Similarly, the assessment of cancer-testis antigen is valuable in evaluating treatment response in lung cancer patients. A survival analysis within an immunotherapy cohort of NSCLC patients indicated that a high cancer testis antigen burden in patients treated with pabolizumab monotherapy correlated with longer survival times [187].

Moreover, a study by Nikolaus and colleagues assessed the performance of HMGB1 against well-established tumor markers such as CYFRA 21 − 1, CEA, and NSE. The results showed that HMGB1 correlated with a reduced overall survival in NSCLC patients, suggesting its capability as a predictive marker for treatment outcomes and prognosis in advanced NSCLC cases [188]. These findings emphasize the reliability of TAAs as tumor markers, proving their value in tracking treatment efficacy and predicting clinical outcomes in lung cancer patients.

### Prognostic assessment, recurrence, and metastasis monitoring

Accurate prognostic assessment is crucial in determining the appropriate treatment strategy for patients with surgically resectable lung cancer. Ideally, a biomarker for the prognostic evaluation should possess both high sensitivity and specificity, although finding markers that satisfy both criteria can be challenging. The selection of markers for the diagnosis and prognostic evaluation of lung cancer should aim to achieve a balance between high sensitivity and specificity to meet various clinical needs and goals [189].

CTCs and ctDNA hold significant potential in the prognostic assessment and surveillance of metastatic recurrence in lung cancer patients. These biomarkers primarily predict patients’ survival prognosis and trends in metastatic recurrence, aiding in the development of personalized clinical strategies [190]. Previous studies have demonstrated that CTC counting not only allows for the early diagnosis of lung cancer but also that specific subgroups of CTCs can reflect certain aspects of the tumor’s status, including its metastatic tendencies [191]. For instance, the counts of total CTCs and vimentin-positive (vim^+^) CTCs in lung cancer patients can be used to assess clinical features, tumor genotype, and survival rates [192]. The spatial distribution of CTCs was found to be heterogeneous, and CTC counts were predictive of 3- and 5-year survival in lung cancer patients [193]. In addition, the detection of CTCs and plasma metabolite profiling may aid in diagnosing early-stage lung cancer and identifying patients at risk of disease recurrence [124]. Increased CTCs in patients undergoing radical resection for lung cancer may predict an elevated risk of tumor recurrence [194]. Detecting and characterizing minimal residual disease, a potential risk factor for recurrence after tumor treatment, can be achieved by analyzing CTCs, offering insights into the risk of recurrence and metastatic dissemination of lung cancer [195, 196]. The TLR4-ERK5 axis has been suggested as a potential biomarker for disease recurrence and metastasis in lung cancer, as it is associated with the aggregation of CTCs and contributes to treatment failure and distant metastasis [197]. Notably, tumor recurrence and metastasis can increase the number of CTCs in the blood, possibly due to the continued release of CTCs from metastatic tumors to support tumor proliferation and invasion [198]. Thus, characterization and analysis of CTCs can help to understand the prognosis and monitor recurrence in lung cancer patients.

Currently, ctDNA research is focused on analyzing multiple mutation information, including EGFR, ALK, and KRAS, to assess the prognosis and disease recurrence of lung cancer patients [199]. Detecting EGFR mutations in lung cancer using ctDNA allows for the assessment of various clinical information, such as the response to ositinib therapy, survival time, and the risk of distant metastasis in lung cancer [200]. Perioperative ctDNA analysis can detect MRD of resectable NSCLC earlier than conventional radiological imaging, providing a means to monitor tumor recurrence and metastasis [201]. Elevated ctDNA levels in the blood of lung cancer patients may predict tumor metastasis, and analyzing and characterizing lung cancer ctDNA can explore tumor mutation loads, genotypes, and genotype-specific differences, contributing to the surveillance of NSCLC recurrence and risk assessment for metastasis [202, 203]. Importantly, ctDNA not only monitors lung cancer recurrence and metastasis but also offers new avenues for ctDNA-driven therapeutic research by characterizing the developmental trajectory of lung cancer [204].

Previous studies have confirmed the role of non-coding RNA (including miRNA, lncRNA, and circRNA) in prognostic assessment and tumor metastasis in lung cancer. Many of these studies utilized multifactorial predictive risk models to assess the prognosis and metastasis of lung cancer through survival analysis and tumor TMN staging [205]. Dai et al. [206] constructed a lung cancer prognostic model comprising eight miRNAs (miR-1260b, miR-21-3p, miR-92a-3p, miR-2467-3p, miR-4659a-3p, miR-4514, miR-1471, and miR-3621) to evaluate the prognosis and metastatic risk of lung cancer. Similarly, prognostic models constructed by lncRNA and circRNA can be used for the analysis and evaluation of clinical features related to prognosis and metastasis in lung cancer [207]. Three circRNAs (hsa_circ_0062682, hsa_circ_0092283, and hsa_circ_0070610) are considered potential targets for prognostic analysis of lung cancer [208], and a lung cancer prediction model composed of nine lncRNAs effectively analyzes metastatic risk, prognostic outcomes, and clinicopathological features of lung cancer [209]. These results suggest the potential clinical application of non-coding RNA as a liquid biopsy for prognostic assessment and metastasis monitoring in lung cancer.

For EVs, Jin et al. evaluated the potential of EVs carrying WASL, STK10, and WNK1 for lung cancer diagnosis and prognostic assessment [210]. They concluded that low expression of STK10 and WNK1 proteins in the EVs of lung cancer patients correlated with a good prognosis of lung cancer compared to the healthy population. In addition, EVs of lung cancer origin enable the characterization of a variety of substances and may contribute to the generation of an immunosuppressive microenvironment and tumor proliferation. For example, high expression of PD-L1 in EVs inhibits CD8^+^ T cell activity to promote lung cancer cells to evade the immune system and invade neighboring tissues [211]. Moreover, a study suggested that analyzing PD-L1 carried by EVs in NSCLC patients treated with immune checkpoint inhibitors is a potential strategy for predicting treatment outcomes and prognosis in cancer patients [212]. Besides PD-L1/PD-1, other immune checkpoints associated with EVs and capable of analyzing the prognostic characteristics of lung cancer are also of interest. High expression of CD155/TIGIT has been suggested to be potentially associated with tumor immune escape and a poor prognosis [213, 214]. Notably, the simultaneous use of multiple biomarkers may offer more benefits than relying on a single biomarker. This approach not only enhances the diagnosis and monitoring of lung cancer recurrence but also aids in the early diagnosis of hidden metastatic lesions in lung cancer.

Standard metabolic programs play a crucial role in the proliferation and metastasis of tumors. Analyzing clinical information, such as prognosis and metastasis in lung cancer, can be facilitated by studying the expression of metabolism-related genes. Wang et al. [215] developed a prognosis-related model using these genes and identified PSMC6, SMOX, and SMS as independent prognostic factors for lung cancer. They also examined the clinical outcomes of lung cancer patients, revealing associations between metabolism-related genes, immune cell infiltration, and immunotherapy effects. Moreover, glutamine metabolism-related genes have been proposed as indicators for assessing the prognosis of lung cancer patients, with LGALS3 identified as a potential therapeutic target [216]. In addition to prognostic models, elevated levels of metabolites in the blood or tissues of lung cancer patients may signify tumor progression. Monitoring these metabolite changes not only aids in evaluating patient prognosis but also in dynamically tracking metastasis and recurrence [7]. For example, a high expression of pyrimidine metabolism rate-limiting enzymes in the blood is a poor prognostic factor for lung adenocarcinoma and may promote lung cancer metastasis [217].

The use of TEP RNA as a liquid biopsy holds great promise in revolutionizing tumor prognosis, recurrence monitoring, and metastasis assessment as a minimally invasive method to complement traditional tissue biopsies. TEP RNA is particularly advantageous in lung cancer patients, addressing challenges posed by the inaccessibility of tumor sites or patients’ poor physical condition hindering tolerance for a tumor biopsy [218]. Ge et al. [219] identified seven TEP liquid biopsy biomarkers for tumor prognostic assessment and metastasis surveillance. In a separate study, linc-GTF2H2-1 and RP3-466P17.2 were significantly down-regulated, while lnc-ST8SIA4-12 was significantly up-regulated in TEPs from lung cancer patients compared to healthy donors (with AUCs of 0.781, 0.788, and 0.725). This offers a potential liquid biopsy approach using blood-derived TEPs for diagnosing and predicting the progression of lung cancer [220]. Similarly, tumor-educated platelet integrin α2b has been proposed as a valuable indicator for prognostic evaluation in NSCLC [148]. Thus, TEPs have emerged as a promising biomarker for the diagnosis and prognosis of lung cancer, with further research needed to fully unlock its potential as a liquid biopsy biomarker [93].

Given the presence of TAAs in the body fluids of lung cancer patients, leveraging TAAs for monitoring tumor recurrence and metastasis has emerged as a promising strategy. A comprehensive characterization of the expression levels of TAAs in the body fluids of lung cancer patients is essential for effectively tracking events such as postoperative recurrence and tumor metastasis. The tumor-associated antigen L6 (TAA-L6), which is expressed in the majority of epithelial cell carcinomas, acts as a target for antibody-mediated treatments. Notably, elevated expression of TAA-L6 has been associated with an increased risk of early postoperative recurrence (*P* = 0.034) and decreased survival rates (*P* = 0.025) in patients with squamous cell lung cancer [221]. In a study conducted by Jillian A. et al. [222], a liquid biopsy approach was used to assess PD-L1 expression on tumor-associated cells to track lung cancer recurrence and metastasis in patients undergoing treatment with immune checkpoint inhibitors. The study found that a significant increase in PD-L1 expression was associated with a better prognosis, indicating a lower likelihood of lung cancer recurrence and metastasis.

Additionally, the tumor-associated antigen MAGE-B2, identified during serum sequencing of lung cancer patients, proved effective in liquid biopsy monitoring. Preoperative observations showed high antibody titers against MAGE-B2, which decreased following the surgical removal of the primary tumor. These titers then increased in the presence of adrenal metastases and decreased once again after the metastatic tumors were surgically removed [223]. This study affirms MAGE-B2’s efficacy as a TAA in liquid biopsies for monitoring lung cancer recurrence and metastasis. It is crucial to note that biomarkers with both specificity and sensitivity must be carefully selected for the identification and differentiation of TAAs in liquid biopsies. This caution arises from the fact that some TAA expressed in tumors may also be present in the healthy population. While combining multiple TAAs for monitoring tumor recurrence and metastasis could improve accuracy, this strategy poses potential challenges in antigen identification and utilization of liquid biopsy techniques.

### Liquid biopsies and lung cancer immunotherapy

Tumor immunotherapy is designed to activate the body’s immune response against tumors by boosting the immunogenicity of tumor cells and making them more vulnerable to destruction by effector cells. Within the realm of lung cancer immunotherapy, liquid biopsy has emerged as a valuable tool for evaluating and monitoring treatment efficacy.

A substantial body of evidence-based medical data now supports the prognostic assessment of lung cancer patients and the evaluation of immunotherapy effectiveness, particularly through the promising clinical applications of CTCs. In a clinical study involving 104 lung cancer patients, CTCs were detected in the blood of one-third of the patients. Their presence independently indicated an absence of a sustained response to immune checkpoint inhibitors (ICIs) and was associated with shorter progression-free survival and overall survival rates [224]. Consistent findings from other studies suggest that an increased number of CTCs in the blood may signal a less favorable response to ICI treatment and a poorer survival outcome [225]. Furthermore, CTCs in liquid biopsies provide valuable information about PD-L1 expression. Numerous studies have explored the effects of PD-L1-positive CTCs on the clinical outcomes of lung cancer patients receiving ICIs. Specifically, elevated PD-L1 expression in patients treated with natalizumab correlated with worse outcomes, and PD-L1-positive CTCs were identified in all patients who had received immunotherapy [226].

Furthermore, the observed increase in PD-L1-positive CTCs during immunotherapy indicates a potential development of resistance to immune checkpoint inhibitors in tumor patients [227]. However, conflicting studies have presented divergent views, asserting that PD-L1 expression in CTCs does not significantly affect the prognosis of lung cancer immunotherapy, and PD-L1 expression in tissues remains independent of CTCs [227, 228]. This contradiction could be due to the absence of standardized procedures for measuring PD-L1 expression, underscoring the need for additional research to establish uniform methods for detecting CTCs and analyzing PD-L1 antibodies. Continued investigation in this area is expected to enhance our understanding of how PD-L1 expression on CTCs interacts with the effectiveness of lung cancer immunotherapy.

This study assesses the capability of circulating-free DNA (cfDNA) levels in the blood as a predictive marker for clinical outcomes in patients undergoing treatment with ICIs. The findings reveal a compelling association between lower cfDNA levels and improved clinical outcomes with NSCLC immunotherapy [229]. However, the application of total cfDNA levels as a biomarker for lung cancer requires further validation due to the variability in cfDNA production across different lung cancer subtypes.

Circulating tumor DNA (ctDNA), on the other hand, has shown promise as a valuable alternative by providing information on the actual tumor burden and disease-specific genomic status. It acts as a prognostic biomarker for treatment with ICIs, where significant decreases in ctDNA levels are associated with benefits from immunotherapy and longer survival in lung cancer patients receiving anti-PD-1/PD-L1 therapies [230]. Moding et al. [231] presented evidence that the presence of detectable ctDNA after chemotherapy in lung cancer patients was linked to significantly better prognoses when followed up with consolidation therapy using ICIs.

Conversely, higher ctDNA levels early in the chemotherapy suggested a poorer prognosis for patients. Furthermore, ctDNA analysis through liquid biopsy can identify point mutations linked to immunotherapy sensitivity, aiding in the identification of patients who may derive greater benefits from ICIs. This, in turn, guides personalized immunotherapy strategies. The detection of co-mutations in STK11/KRAS has been recognized as an indicator of worse survival outcomes in patients undergoing treatment with ICIs [232], reinforcing the viability of detecting mutation sites associated with immunotherapy to predict its effectiveness.

Extracellular vesicles (EVs) offer a promising approach for assessing the efficacy of tumor immunotherapies. In research analyzing PD-L1 mRNA expression in EVs from NSCLC patients undergoing treatment with pembrolizumab and natalizumab, it was found that PD-L1 mRNA levels were notably higher in those who responded to treatment compared to those who did not, prior to the start of immunotherapy. After two months of treatment, individuals who responded showed a significant decrease in PD-L1 mRNA copy numbers, in contrast to non-responders, who experienced an increase. This indicates that measuring PD-L1 levels in plasma EVs could serve as an effective method for evaluating the success of immunotherapy [233]. In a study involving 21 advanced NSCLC patients, a fold increase of PD-L1 expression ≥ 1.86 in EVs was associated with enhanced efficacy of immunotherapy and extended survival [234]. This suggests that an increase in PD-L1 mRNA and/or PD-L1 protein levels in EVs during ICI therapy could act as a beneficial biomarker for both the effectiveness and prognosis in advanced NSCLC patients. Moreover, the genetic content within EVs, including essential non-coding RNAs, is critical for evaluating the success of lung cancer immunotherapy. Peng et al. investigated the role of miRNAs in plasma EVs as indicators of the therapeutic effects of ICIs. Three miRNAs (has-miR-320b, has-miR-320c, and has-miR-320d) were identified as predictors for assessing response to treatment, and has-micro-125b-5p was highlighted as a potential target for enhancing anti-PD-1 therapy [235]. Regular tracking of has-miR-125b-5p levels is recommended as a strategy during anti-PD-1 therapy, particularly for patients who show a delayed response or experience pseudoprogression. This approach aims to provide a more accurate assessment of treatment efficacy and guide adjustments in therapy for those not immediately showing clear signs of improvement. Alterations in specific circulating miRNAs have been observed to influence both the response to treatment and survival rates during ICI therapy in NSCLC patients. In a cohort study of NSCLC patients treated with immunotherapy, researchers identified 27 miRNAs with differential expression patterns—22 were found to be highly expressed, and five showed low expression. Further validation in a separate group of patients confirmed that the upregulation of 10 miRNAs was positively correlated with better outcomes from immunotherapy and prolonged survival, highlighting the potential of these miRNAs as biomarkers for predicting the efficacy of ICI treatments [236]. Additionally, another study introduced a miRNA signature classifier comprising 24 miRNAs that successfully differentiated NSCLC patients who responded favorably to anti-PD-L1 immunotherapy from those who did not benefit [237]. These results suggest the potential of EVs and non-coding RNAs, particularly miRNAs, in predicting immunotherapy responses in NSCLC patients in a non-invasive manner in lung cancer. Moreover, the exploration of other non-coding RNAs, such as circular RNAs and lncRNAs, holds equal promise in contributing to our understanding of lung cancer and refining predictive markers for immunotherapy outcomes.

The investigation of plasma metabolites as potential targets for prognostic assessment and metastasis monitoring in lung cancer patients presents an intriguing avenue for informing immunotherapy strategies. A study focusing on refractory large B cell lymphoma identified six markers, such as acetylspermidine, diacetylspermidine, and various lysophospholipids, through metabolomics analysis of liquid biopsies. This research, which concentrates on polyamines, suggests that the levels of these metabolites can predict responses to CAR-T cell therapy. Specifically, elevated levels of these metabolites may indicate a lower likelihood of a positive response to CAR-T cell treatment, offering valuable insights into patient stratification and the potential customization of therapeutic approaches [238]. Additionally, an increase in amino acid metabolites, specifically indoleamine 2,3-dioxygenase 1, in ascites of ovarian cancer patients suggested the ineffectiveness of immunotherapy in these individuals [239]. While these studies are indirectly related to lung cancer immunotherapy, they are a good stimulus for guiding immunotherapy research in lung cancer. The efficacy of metabolites in lung cancer immunotherapy remains to be uncovered in future investigations.

The current literature regarding TEP in lung cancer immunotherapy is limited. However, available evidence points to an increase in PD-L1 expression within TEP among patients with lung cancer. Intriguingly, administration of atezolizumab, a PD-L1 targeting monoclonal antibody, has been observed to lead to a reduction in PD-L1 expression in TEP. This change suggests TEP’s potential role in tracking responses to immune-based treatments in lung cancer [240]. For TAAs, studies by Anne et al. revealed correlations between different immune cell populations in lung cancer and immunopeptidomes. A notable discovery was the higher frequency of predicted neoantigens at HLA-I presentation hotspots in tumors where CD3 + CD8 + T cells were not present, indicating a possible connection between these neoantigens and immune recognition. This relationship could influence the process of immunoediting, affecting tumor mutations and the efficacy of immunotherapy in lung cancer patients [241]. Moreover, employing TAA-specific T-cell receptor (TCR) gene-modified T cells has shown considerable promise in anti-tumor immunotherapy, with hematopoietic lineage-converted T cells armed with TCRs targeting TAAs demonstrating effective tumor cell elimination capabilities [242]. These findings demonstrate the potential of TEP and TAA in lung cancer immunotherapy, although further studies are essential for the validation and confirmation of these potential therapeutic avenues.

## Conclusions and future prospects

Lung cancer represents a significant menace to human health, emerging as the foremost cause of cancer-related fatalities [243]. Many individuals are diagnosed at advanced tumor stages, precluding the possibility of surgical intervention, with radiotherapy and chemotherapy being the primary conservative treatment choice. The challenges of chemotherapy resistance and radiation-induced damage further compound the prognosis dilemma for patients. Even post-surgical resection and radio-chemotherapy, there remains a risk of tumor recurrence and metastasis, as the accuracy of imaging results in assessing prognosis and recurrence is not always reliable [244]. Therefore, there is an urgent need for new biological tools to ameliorate the present difficulties.

Liquid biopsies, as a groundbreaking biomedical testing method, exhibit promising advantages over traditional tissue biopsies in terms of affordability and convenience, particularly when physical constraints impede the acquisition of tissue specimens. By detecting relevant biomarkers released by tumor cells during progression, liquid biopsies offer insights into tumor heterogeneity, thereby enhancing the accuracy of tumor diagnosis and prognosis assessment. Liquid biopsies allow clinicians to continuously sample and dynamically observe tumors and make informed decisions based on tumor progression to improve clinical treatment strategies. Additionally, the application of liquid biopsy involves the comprehensive evaluation and monitoring of lung cancer patients for recurrence and metastasis. This encompasses the development of more favorable clinical protocols, particularly beneficial for surgically treated patients and those who missed the optimal treatment window. This aspect is particularly crucial in high-fatality diseases like lung cancer.

Nevertheless, the preclinical application of liquid biopsy faces several challenges. The technology for characterizing biomarkers through liquid biopsy is still in a nascent stage, characterized by complex processes and inefficient equipment that hinder its full development. Detecting biomarkers, particularly low levels of ctDNA and EVs in the blood of oncology patients, remains a challenge, emphasizing the need to improve detection efficiency for the advancement of liquid biopsy. Digital PCR (dPCR) stands out for its high sensitivity, making it adept at detecting rare variants and subtle copy number variations. Its reproducibility is noteworthy, allowing for easy calculation of performance metrics and error rates through mathematical equations, thereby facilitating workflow optimization. Moreover, dPCR is stable, making it a valuable tool for validating other techniques. The simplicity of its workflow reduces bench time and overall costs. However, hurdles hinder its routine clinical application, such as that (i) dPCR requires perfect pre-analytical and analytical procedures, (ii) the standardization of samples is still an obstacle that must be overcome, (iii) and the limited number of detectable targets, necessitating prior next-generation sequencing (NGS) testing for comprehensive assessment. NGS technology is another commonly used liquid biopsy technology, offering comprehensive information on individual gene mutations in ctDNA, CTCs, and EVs. This capability aids in overcoming tumor heterogeneity, allowing for a comprehensive analysis of the tumor mutation landscape and facilitating clinical monitoring of tumor mutation load. Despite its advantages, challenges impede NGS’s full clinical potential. The intricacies of NGS library construction and the lack of consensus on sample standardization pose hurdles, potentially limiting sensitivity and accuracy. Collaborative efforts, including industry, technicians, and clinicians, are crucial for realizing the clinical application of NGS. Addressing the following issues, including continuous optimization and expediting database construction and analysis process, building drug-mutation correlation databases, implementing strict and standardized evidence grading for clinical interpretation, and fostering close collaboration between detection and treatment, will propel the development of NGS technology. These measures are vital for creating industry consensus and a shared knowledge base, ultimately benefiting patients.

Additionally, to comprehensively understand the complex features of tumors and mitigate individualized differences, it is essential to integrate other diagnostic tests such as CT, MRI, and ultrasound with liquid biopsy results. Recognizing that biomarkers in body fluids from various sources may reflect different tumor characteristics. For instance, urine may reveal characteristics of bladder tumors, while cerebrospinal fluid may respond to brain tumor progression. In the context of lung cancer, focusing on pleural fluid may prove more effective. Furthermore, while numerous liquid biopsy-based biomarker models have emerged from data analyses, their accuracy in prognostic assessment and metastasis surveillance in lung cancer patients requires confirmation through additional experiments. As ctDNA, CTCs, EVs, TEPs, and other assays undergo refinement, their potential as robust biological tools for various aspects of lung cancer management becomes increasingly evident. These advancements hold promise for improving early diagnosis, monitoring treatment responses, and assessing the recurrence and metastasis of lung cancer. In essence, the evolution of liquid biopsy represents a significant stride in the realm of precision medicine, and it stands poised to transition from a promising concept to a tangible clinical reality in the future.

## Data Availability

Not applicable.

## Abbreviations

ARMS: Amplification refractory mutation system
CEA: Carcinoembryonic antigen
CE-MS: Capillary electrophoresis-mass spectrometry
CTCs: Circulating tumor cells
ctDNA: Circulating tumor DNA
DDA: Data-dependent acquisition
ddPCR: Droplet digital PCR
dPCR: Digital PCR
ECAM: Epithelial cell adhesion molecules
EMT: Epithelial-mesenchymal transition
EUS-FNA: Endoscopic ultrasonography-guided fine-needle aspiration
EVs: Extracellular vesicles
GC-MS: Gas chromatography mass spectrometry
ICIs: Immune checkpoint inhibitors
LC-MS: Liquid chromatography mass spectrometry
LDCT: Low-dose spiral CT
LUAD: Lung adenocarcinoma
LUSC: Lung squamous cell carcinoma
miRNAs: microRNAs
MRD: Minimal residual disease
MRI: Magnetic Resonance Imaging
MRM: Multiple reaction monitoring
MS: Mass spectrometry
ncRNAs: Non-coding RNA
NGS: Next generation sequencing
NMR: Nuclear magnetic resonance
NSCLC: Non-small cell lung cancer
qRT-PCR: Quantitative real-time PCR
SCLC: Small cell lung cancer
TAAs: Tumor-associated antigens
TEP: Tumor-educated platelets
TKIs: Tyrosine kinase inhibitors
